# Direct Co-Targeting of Bcl-xL and Mcl-1 Exhibits Synergistic Effects in AR-V7–Expressing CRPC Models

**DOI:** 10.1158/2767-9764.CRC-25-0096

**Published:** 2025-08-21

**Authors:** Benjamin C. Brim, Andres F. Leon, Erica L. Beatson, Jessica D. Kindrick, Kinjal Bhadresha, Xiaohu Zhang, Giulia C. Napoli, Emily N. Risdon, Keith T. Schmidt, Kelli M. Wilson, Crystal McKnight, Erin Beck, Carleen Klumpp-Thomas, Michele Ceribelli, JuanJuan Yin, Adam G. Sowalsky, Douglas K. Price, Cindy H. Chau, Craig J. Thomas, William D. Figg

**Affiliations:** 1 Molecular Pharmacology Section, Genitourinary Malignancies Branch, Center for Cancer Research, National Cancer Institute, National Institutes of Health, Bethesda, Maryland.; 2 Division of Preclinical Innovation, National Center for Advancing Translational Sciences, National Institutes of Health, Rockville, Maryland.; 3 Clinical Pharmacology Program, Center for Cancer Research, National Cancer Institute, National Institutes of Health, Bethesda, Maryland.; 4 Prostate Cancer Genetics Section, Genitourinary Malignancies Branch, Center for Cancer Research, National Cancer Institute, National Institutes of Health, Bethesda, Maryland.

## Abstract

There is an unmet need to develop novel treatment options for patients with metastatic castration-resistant prostate cancer (mCRPC). Patients often develop resistance to next-generation hormonal therapies that target the androgen receptor (AR) axis (e.g., abiraterone and enzalutamide). A splice variant of AR, AR-V7, is associated with resistance to these inhibitors as well as mCRPC progression and poor prognoses. We embarked upon a high-throughput screen to identify synergistic combinations of targeted therapies using two CRPC cell lines, LNCaP95 and VCaP-CR. Combinations targeting BCL2L1 (Bcl-xL) (A-1331852 and navitoclax) and MCL1 (S63845) synergistically decreased cell viability and induced apoptotic activity via cleavage of PARP, caspase 3, and caspase 7 across AR-V7–expressing CRPC cell lines (LNCaP95, VCaP-CR, and 22Rv1) and a patient-derived organoid model (LuCaP 167CR). We also explored the use of a Bcl-xL–specific proteolysis-targeting chimera degrader (PROTAC) to minimize platelet toxicity associated with Bcl-xL inhibitors. We showed similar synergistic efficacy with the Bcl-xL–targeting PROTAC in combination with S63845 in the three-dimensional spheroid models. Our findings support further preclinical development of Bcl-xL and Mcl-1 inhibitors for mCRPC.

**Significance:**

Using an unbiased, combinatorial, high-throughput drug screen, we identified the combination of co-targeting Bcl-xL and Mcl-1 to be highly synergistic across AR-V7–expressing CRPC models. We showed efficacy in higher-order models through validation across *in vitro* models spanning two-dimensional cell culture, three-dimensional cell culture, and a patient-derived organoid model. These findings identify a promising therapeutic strategy for patients with AR-V7–expressing CRPC.

## Introduction

Overcoming acquired resistance following the treatment of metastatic castration-resistant prostate cancer (mCRPC) is an unmet clinical need despite the availability of numerous life-prolonging therapies (1). Several pathways are implicated in therapeutic resistance, with many involving aberrations to the androgen receptor (AR), especially the constitutively active AR splice variant AR-V7 (2). AR-V7 lacks the ligand-binding domain of AR, allowing for activation of the AR signaling pathway in the absence of androgens, and is associated with progression to mCRPC and poor prognoses (3). AR-V7 has also been implicated in resistance to novel hormonal therapies that target the AR axis (e.g., abiraterone and enzalutamide; refs. 2, 4, 5). Since AR-V7 plays a critical role in CRPC progression and the emergence of resistance, this provides impetus to develop new therapeutics that can overcome AR-V7–mediated treatment resistance.

Evasion of apoptosis is a hallmark of cancer (6), allowing malignant cells to proliferate continuously. Pro- and anti-apoptotic proteins regulate the intrinsic (mitochondrial) apoptosis pathway. The anti-apoptotic proteins, specifically BCL2, BCL2L1 (Bcl-xL), and MCL1, are often overexpressed in cancer (7) and are associated with resistance and disease progression in prostate cancer (8, 9). BH_3_ mimetics, a class of small-molecule antagonists of the anti-apoptotic Bcl-2 family of proteins, have been developed to induce apoptosis in Bcl-2 family–dependent cancer cells (10). Direct co-targeting of Bcl-xL and Mcl-1 in cervical cancer, melanoma, and medulloblastoma has been shown to synergistically reduce cell viability (11–13). Additionally, indirect downregulation of Mcl-1 was shown to sensitize prostate cancer cells to navitoclax (14). Taken together, the direct co-targeting of Bcl-xL and Mcl-1 in AR-V7–expressing CRPC preclinical models is an exciting combination worth exploring.

We performed a high-throughput drug screen using AR-V7–expressing CRPC cell lines (LNCaP95 and VCaP-CR) to identify synergistic drug combinations. Synergism allows for antitumor activity while reducing required doses, limiting potential toxicities. We identified the combination of direct inhibitors of Bcl-2 family proteins through BH_3_ mimetics to be highly synergistic. Expanding upon this, we hypothesize that combined and direct co-targeting of Bcl-xL and Mcl-1 will synergistically reduce cell viability while inducing apoptosis. The current study aims to optimize the dual targeting of these two Bcl-2 family proteins across several AR-V7–expressing two-dimensional (2D)/three-dimensional (3D) cultures and organoid models while attempting to minimize doses to avoid potential toxicities.

## Materials and Methods

### Cell culture and reagents

The prostate cancer cell line 22Rv1 (RRID: CVCL_1045) was purchased from the ATCC. LNCaP95 (RRID: CVCL_ZC87) and VCaP-CR cell lines (RRID: CVCL_A0BX) were kindly provided by Dr. Jun Luo (Johns Hopkins Biobank). Unless otherwise specified, cell culture reagents were purchased from Gibco/Thermo Fisher Scientific. 22Rv1 cells were maintained in RPMI-1640 supplemented with 10% FBS (Premium Select FBS, R&D Systems,), 50 U/mL penicillin, and 50 mg/mL streptomycin (1%, Gibco). LNCaP95 cells were maintained in phenol red–free RPMI-1640 supplemented with 10% charcoal dextrin–stripped FBS (R&D Systems) and 1% penicillin/streptomycin on Cell+ cell culture flasks (Sarstedt). VCaP-CR cells were grown in phenol red–free RPMI-1640 supplemented with 10% charcoal dextrin–stripped FBS and 1% B-27 neuronal supplement (Gibco) on Cell+ cell culture flasks. Cells were maintained at 37°C in an atmosphere containing 5% CO_2_ and 95% humidity. Cell line authentication was routinely performed by short tandem repeat genotyping (ATCC/Labcorp). *Mycoplasma* contamination testing was routinely performed by the MycoAlert Mycoplasma Detection Kit (Lonza, LT07-318). Cells were used for no longer than 3 months after being thawed.

### Compounds

BH_3_-mimetic drugs [navitoclax (ABT-263), venetoclax (ABT-199), A-1331852, and S63845] were purchased from Selleckchem. XZ739 was purchased from MedChemExpress. All drugs were prepared as 10 mmol/L stock solution in DMSO, aliquoted, and freshly diluted in the appropriate media immediately before use to yield a final DMSO concentration of ≤0.5% for each experiment.

### High-throughput drug screen

For pairwise drug combination assessments in the matrix format, compounds were acoustically dispensed into a 1536-well white solid-bottom tissue culture–treated plate (EWB041000A, Aurora Microplates) with an Echo 550 acoustic liquid handler (Labcyte) using previously described methods (15, 16). A nine-point custom concentration range with 1:2 dilution between points was used for each drug pair tested. The proteasome inhibitor bortezomib (final concentration 20.3 μmol/L) was used as a positive control, and a DMSO negative control was also included within each assay plate. LNCaP95 or VCaP-CR cells were seeded into compound-containing plates at a density of 500 cells/well in a final volume of 5 μL growth media using a Multidrop Combi dispenser (Thermo Fisher Scientific) using a 10 × 10 matrix. The plates were covered by a stainless-steel gasket lid to prevent evaporation and incubated for 48 hours (LNCaP95) or 72 hours (VCaP-CR) in a humidified CO_2_ incubator. At the readout time point, 3 μL of CellTiter-Glo (Promega) was added to each well using a BioRAPTR (Beckman Coulter), and the plates were incubated at room temperature for 15 minutes with the stainless-steel lid in place. Luminescence readings were taken using a ViewLux reader (PerkinElmer) with a 2-second exposure time per plate. The viability of compound-treated wells was normalized to DMSO, empty-well controls were present on each plate, and combination–response plotting was automatically performed for each drug–drug combination. DBSumNeg, a synergy score, was calculated for each pairing (17).

### CellTiter-Glo luminescent cell viability assay

Cells were seeded onto clear-bottom polystyrene or amine-coated black 96-well plates (15,000 for 22Rv1s and LNCaP95 and 20,000 for VCaP-CRs) and allowed to incubate for 24 to 48 hours at 37°C in 5% CO_2_. The media were then replaced with treatment media containing vehicle or test compounds at a range of concentrations alone or in combination for 48 hours (LNCaP95 and 22Rv1) or 72 hours (VCaP-CR) of incubation using a 6 × 8 matrix. Cell viability was assessed using CellTiter-Glo (Promega) according to the manufacturer’s instructions, and luminescence was measured on a SpectraMax iD3 plate reader (Molecular Devices). All individual readings were normalized to the average of the DMSO vehicle control group and then analyzed using GraphPad Prism 8.4.2 (GraphPad, RRID: SCR_002798) to generate IC_50_ curves. Drug combination synergy calculations were performed using the SynergyFinder+ R-based package (RRID: SCR_019318; ref. 18) and were quantified using the Bliss independence analysis model (19). Synergy is indicated by a mean Bliss value greater than 10. Additivity is defined by a mean Bliss value between −10 and 10. In contrast, antagonism is indicated by a mean Bliss value less than −10.

### Western blot analysis

Cells were seeded onto six-well plates and allowed to incubate overnight at 37°C in 5% CO_2_. The cells were treated with vehicles or compounds for 24 hours. Total protein extracts were prepared by washing cells with ice-cold PBS and then lysing with RIPA lysis buffer (Sigma) and a complete protease inhibitor cocktail (Nacalai). Samples were left on ice for 10 minutes, briefly vortexed, and returned to the ice for another 10 minutes. Lysed cells were then centrifuged at 13,000 × *g* at 4°C for 15 minutes. The protein-containing supernatant was carefully removed, and the amount of protein in the supernatant was quantified using the Pierce Bicinchoninic Acid assay (Thermo Fisher Scientific). Samples were run on 12% SDS-PAGE Mini-PROTEAN TGX Precast Gels (Bio-Rad) and separated by electrophoresis at 80 V for 90 minutes. Gels were transferred to a 0.2-μm nitrocellulose membrane using the Mini Trans-Blot Turbo semi-dry transfer system (Bio-Rad). The nitrocellulose membranes were blocked with 5% nonfat dry milk in TBST containing 0.1% Tween for 60 minutes and probed overnight at 4°C with antibodies against AR (SC-7305; 1:1,000, lot H0521, Santa Cruz Biotechnology, RRID: AB_626671), AR-V7 (RevMAb RM7; 1:1,000, lot U-03–04124, RevMAb Biosciences, RRID: AB_2716436), Bcl-2 (cat. #2876, 1:500, lot 6, Cell Signaling Technology, RRID: AB_2064177), Mcl-1 (SC-12756; 1:200, lot D0318, Santa Cruz Biotechnology, RRID: AB_627915), and Bcl-xL (cat. #2764, 1:1,000, lot 11, Cell Signaling Technology, RRID: AB_2228008). The membranes were washed three times with 0.05% TBST and incubated in goat anti-mouse IRDye 680RD (1:10,000, LI-COR 925-68070, RRID: AB_2651128) and goat anti-rabbit IRDye 800CW (1:10,000, LI-COR 925-32211, RRID: AB_2651127) for 1 hour at room temperature. The membranes were washed three times with TBST containing 0.1% Tween, and blots were imaged on LI-COR Odyssey Fc (LI-COR).

### 3D tumor spheroid assay

3D spheroids were cultured as previously described (20). Spheroids were grown in 96-well U-bottom Ultra-Low Attachment plates (Corning). Cells suspended in 100 μL of fresh medium (750 cells/spheroid and 3,000 cells/spheroid for LNCaP95 and 22RV1s, respectively) were grown for 72 hours without medium change at 37°C at 5% CO_2_. DMSO or test compounds were added to spheroid wells by removing 50 μL of medium and replacing it with 50 μL vehicle- or drug-containing media at 2× concentration. After 72 hours of treatment, cell viability was assessed using CellTiter-Glo 3D Cell Viability Assay (Promega) according to the manufacturer’s instructions, and luminescence was measured on a microplate reader (SpectraMax iD3). Spheroids were imaged daily using the Nikon Eclipse TE2000 U and NIS-Elements software at 4X magnification (1.6125 μm/pixel). Spheroids were measured using ImageJ (RRID: SCR_003070) software to ensure that the diameter was between 200 and 500 μm before treatment. All individual readings were normalized to the average of the DMSO control.

### Organoid drug screening

The patient-derived xenograft model LuCaP 167 used in this study was obtained from the NCI Patient-Derived Models Repository (https://pdmr.cancer.gov; model: K14711-072-R; lot: SB2140). The original LuCaP 167 model was developed at the University of Washington and was derived from a liver metastasis of a 77-year-old Caucasian male resistant to abiraterone, carboplatin, and docetaxel (21, 22). This model expresses the AR and demonstrates castration resistance.

The LuCaP 167CR model was generated from the LuCaP 167 model using previously described methods (21). Briefly, the LuCaP 167 model was implanted in 6- to 7-week-old NOD/SCID gamma (NSG) mice. These mice were castrated using scrotal castration surgery protocol after implantation, and the resulting tumor was harvested and passaged in pre-castrated NSG mice, creating the LuCaP 167CR model.

All mice were operated on under sedation with oxygen and isoflurane. Post-surgery ibuprofen and/or buprenorphine were administered for pain management. Propagation of LuCaP patient-derived xenograft tumors and organoid drug studies were conducted at the NCI under an NCI Animal Care and Use Committee–approved protocol. Tumors were periodically validated through short tandem repeat analysis by Laragen Inc. NCI Animal Care and Use Committee approvals carried out all animal procedures.

For drug screening, tumors were prepared as previously mentioned (22). Briefly, tissue was finely minced and digested in Advanced DMEM with the Gentle-MACS tumor dissociation kit (Miltenyi Biotec, cat. #130-096-427) per kit instructions. The resulting single-cell suspension was centrifuged and passed through 100-μm cell strainers before being treated with red blood cell lysis buffer (Lonza, cat. #10-548E) and then rinsed with DMEM with 10% FBS. Following another filtration with a 70-μm cell strainer, the cells were centrifuged and counted. The cells were either cryopreserved (5 × 10^6^ cells in 90% FBS and 10% DMSO) or seeded for drug screening.

Cells were seeded in a 384-well plate at 2,000 cells per well in 20 μL of 70% growth factor–reduced phenol red–free Matrigel (BD Biosciences, 356231). Prostate organoids were cultured in basal medium containing advanced DMEM/F12 supplemented with 1% penicillin/streptomycin, 1% GlutaMAX (Gibco), 10 mmol/L HEPES (Gibco), 50 × B-27, 5% R-spondin–conditioned medium, 10% Noggin-conditioned media, 5 ng/mL EGF, 100 ng/mL FGF-10, 1 ng/mL FGF-2, 10 μmol/L Y-27632, 10 mmol/L nicotinamide, 1 μmol/L prostaglandin E2, and 1 nmol/L dihydrotestosterone. The next day, organoids were treated with A-1331852, S63845, and/or navitoclax at a range of 10 nmol/L to 5 μmol/L using the Tecan D300e Digital Dispenser (Tecan) and a 7 × 7 matrix. Organoids were retreated with fresh drug-containing media every 3 days. After 10 days, cell viability was measured using CellTiter-Glo 3D per manufacturer’s instructions and luminescence was measured using Infinite M200 PRO microplate reader (Tecan). Individual readings were normalized to the average of the DMSO control. Dose–response curves were generated by fitting the data to a three-parameter logistic regression model with a variable slope, constrained to 100% and 0% viability, using GraphPad Prism 9.0. All cell viability data were collected from three independent biological replicates, each with six technical replicates.

### Patient transcriptomic data

Transcriptome (*n* = 429) data from mCRPC biopsies, generated by the International Stand Up To Cancer (SU2C)/Prostate Cancer Foundation, were downloaded from cBioPortal (23–25) and reanalyzed (26, 27). Cohort expression was displayed utilizing GraphPad Prism, and data are expressed as fragments per kilobase of transcript per million mapped reads.

### Live cell imaging

The Incucyte S3 (Sartorius) was used to track live cell dynamics after treatment with inhibitory compounds. Cells were seeded at 3,000 (LNCaP95 and 22Rv1) or 4,500 (VCaP-CR) cells/well in a black 96-well plate with the Caspase 3/7 live-cell dye (Sartorius, 4440) seeded at a 1:2,000 dilution in a final volume of 100 μL. The cells were allowed to attach for 24 (LNCaP95 and 22Rv1) or 48 hours (VCaP-CR). After attachment, 50 μL of media was removed and replaced with a 2× stock of media treated with DMSO, A-1331852, S63845, or the combination of A-1331852 and S63845. The basic analyzer was used to quantify apoptosis indicated by a green fluorescent signal and the confluency normalized to 0 hour.

### Study approval

All animal work was approved under an NCI Animal Care and Use Committee–approved protocol.

### Statistical analysis

Data are expressed as mean ± SD. Comparisons of spheroid cell viability were made using Mann–Whitney tests. All statistical analyses were performed using GraphPad Prism version 10.4.2, and *P* < 0.05 was used as the threshold for statistical significance.

### Data availability

The data generated in this study are available upon request from the corresponding author.

## Results

### High-throughput screening identifies active synergistic BH_3_-mimetic drug combinations

**Figure 1:**
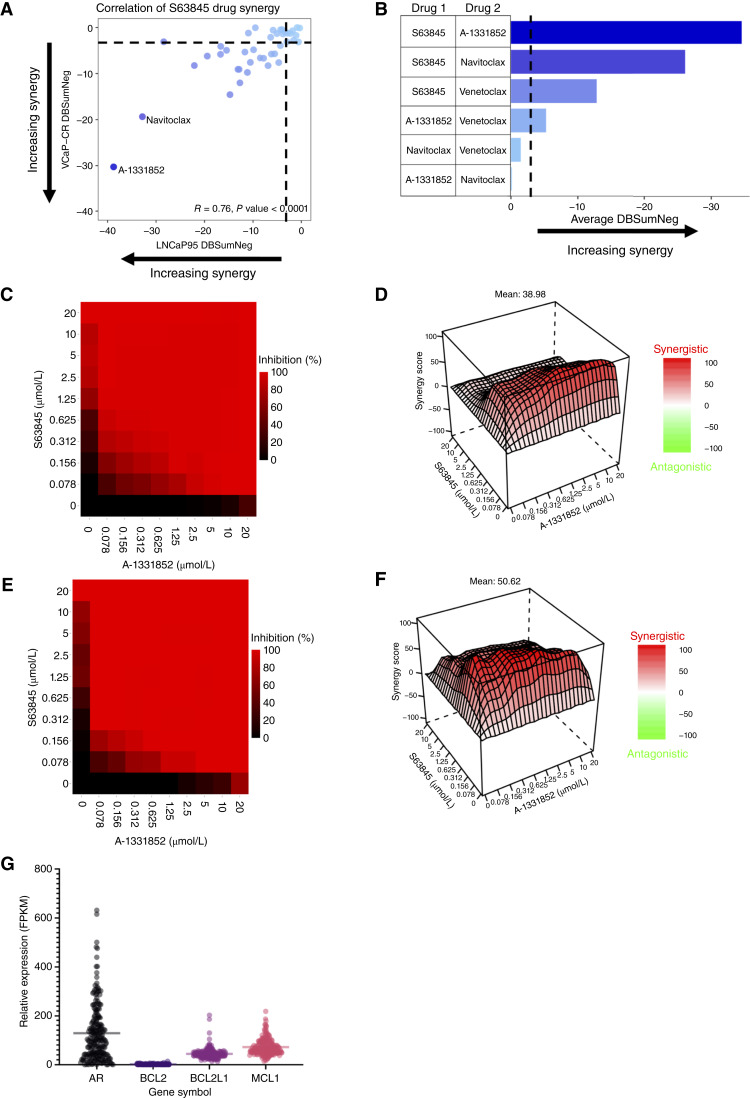
Identification of Synergistic BH_3_-mimetic–based combinations in LNCaP95 and VCaP-CR Cells. **A,** Correlation plot depicting DBSumNeg (synergy score) for drugs that pair well with S63845 across LNCaP95 and VCaP-CR lines. **B,** All pairings of BH_3_ mimetics ranked by average DBSumNeg across LNCaP95 and VCaP-CR lines. **C** to **F,** Example drug screen data from A-1331852 paired with S63845 in VCaP-CR (**C** and **D**) and LNCaP95 (**E** and **F**) cells using CellTiter-Glo to measure response. Cell viability (**C** and **E**) and Bliss synergy were calculated for each point. Z-plots depict Bliss synergy score at each concentration (**D** and **F**). **G,** Patient mRNA sequencing data from the SU2C 2019 cohort of patients with mCRPC reveals the expression of *AR*, *BCL2*, *Bcl-xL*, and *MCL1*. Cohort expression was displayed utilizing GraphPad Prism 10, and data are expressed as fragments per kilobase of transcript per million mapped reads (FPKM).

We evaluated the activity of 2,480 oncology-focused, approved investigational agents from the previously established MIPE 5.0 library (28) utilizing a panel of prostate cancer cell lines (LNCaP, LNCaP95, 22Rv1, PC-3, VCaP, and VCaP-CR). The results indicated numerous classes of drugs with significant single-agent activity, including agents targeting PI3K/AKT, BRD4, and SINE and BH_3_ mimetics (WD Figg/CJ Thomas manuscript in preparation). Next, we conducted 10 × 10 matrix combination screens to identify potential drug synergies in two CRPC cell lines, LNCaP95 and VCaP-CR, using the CellTiter-Glo cell viability assay. Multiple, synergistic drug + drug combinations were discovered, including those involving several pathways of interest for prostate cancer treatment. Among the most synergistic combinations were pairings of approved and investigational drugs targeting the Bcl-2 family isoforms, indicating a potential therapeutic vulnerability for CRPC. The combination of S63845 (Mcl-1 inhibitor) with A-1331852 (Bcl-xL inhibitor) and navitoclax (Bcl-xL/2 inhibitor) demonstrated exceptional synergy in both LNCaP95 and VCaP-CR cells measured by the average DBSumNeg (Fig. 1A). Based on these data, we further examined the combination potential of all possible pairings of BH_3_ mimetics in our primary and confirmation drug-screening efforts (Fig. 1B). Consistently, the combination of S63845 with A-1331852 (Fig. 1C–F) showed the strongest synergy, followed by S63845 and navitoclax and finally S63845 and venetoclax (a selective Bcl-2 inhibitor). Using transcriptomic data from patients with mCRPC in the SU2C/Prostate Cancer Foundation cohort (27), we observed that Mcl-1 and Bcl-xL had higher expression than Bcl-2 (Fig. 1G), indicating that Mcl-1 and Bcl-xL are potential targets in mCRPC.

### Co-inhibition of Mcl-1 and Bcl-xL enhances cytotoxicity in AR-V7–expressing prostate cancer cells

We next evaluated the Mcl-1/Bcl-xL targeting combination in three CRPC cell line models with high AR-V7 expression (LNCaP95, VCaP-CR, and 22Rv1; Supplementary Fig. S1). We first assessed the activity of selective Bcl-2 family protein inhibitors as single agents in LNCaP95, VCaP-CR, and 22Rv1 prostate cancer cell lines (Supplementary Fig. S2; Supplementary Table S1). All three cell lines exhibited limited sensitivity to single-agent BH_3_ mimetics. Overall, the AR-V7–expressing CRPC cell lines were more sensitive to Mcl-1 inhibition than Bcl-xL, Bcl-xL/2, or Bcl-2 inhibition.

**Figure 2:**
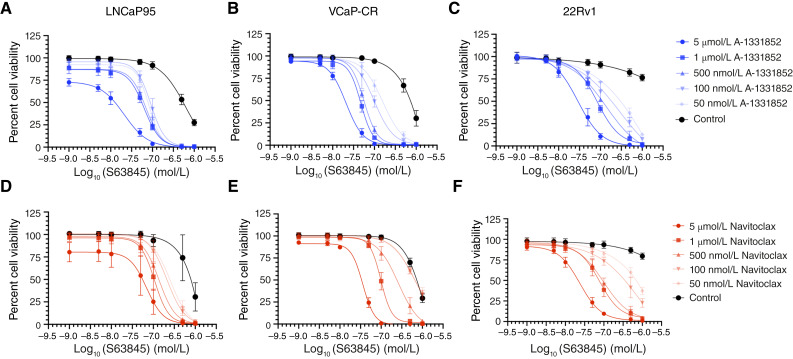
Co-targeting Mcl-1 and Bcl-xL enhances the inhibitory effect on cell viability. The effect on cell viability of A-1331852 (targeting Bcl-xL) in combination with S63845 (targeting Mcl-1; **A–C**) and navitoclax (targeting Bcl-xL and Bcl-2) in combination with S63845 (**D–F**). Cell viability was determined following either 48-hour (22Rv1 and LNCaP-95) or 72-hour treatment (VCaP-CR) by CellTiter-Glo luminescent assay. The data provided represent the mean ± SD from three independent experiments with at least three technical replicates per experiment.

Following the single-agent assessment of sensitivity to Mcl-1 inhibition in prostate cancer cells, S63845 was evaluated in combination with other BH_3_ mimetics to determine synergistic effects on cell viability. Co-treatment with A-1331852 (Fig. 2A–C) or navitoclax (Fig. 2D–F) enhanced the potency of S63845. This led to a leftward shift in the dose–response curve of S63845. A less prominent leftward shift in the dose–response curve of S63845 was seen when treated in combination with venetoclax (Supplementary Fig. S3A–S3C). These results suggest that AR-V7–expressing CRPC cells are more sensitive to co-targeting of Mcl-1 with Bcl-xL rather than Bcl-2. As such, we prioritized the co-inhibition of Mcl-1 and Bcl-xL moving forward.

**Figure 3:**
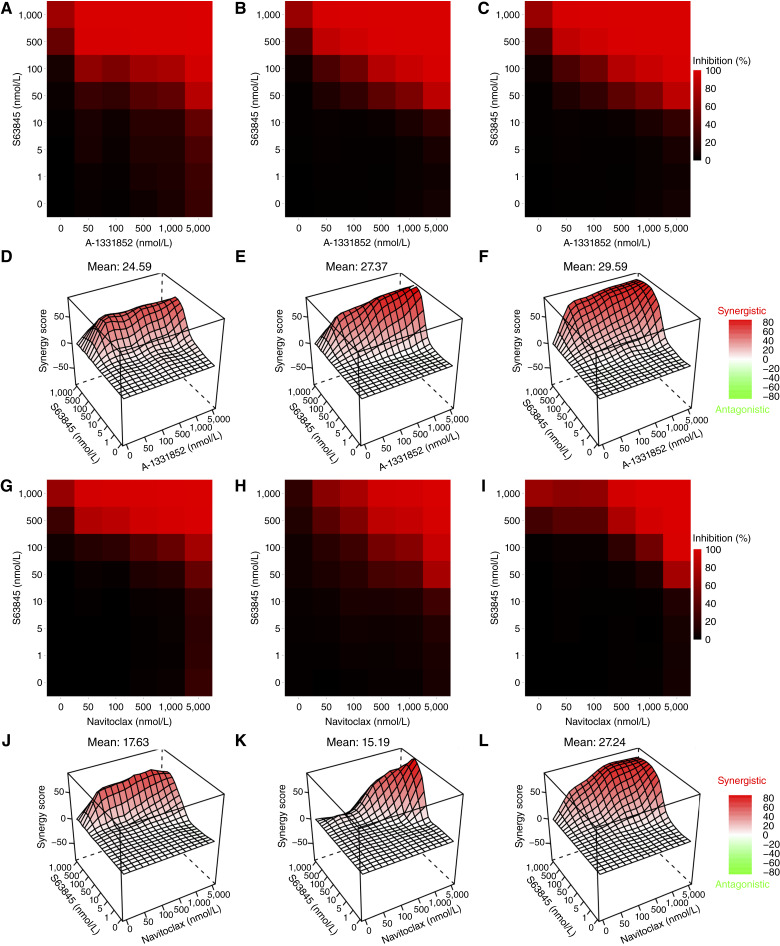
Optimal dose evaluation for co-targeting Bcl-xL and Mcl-1. Dose–response heatmaps (**A–C** and **G–I**) and Bliss synergy plots (**D–F** and **J–L**) for A-1331852-based (**A–F**) and navitoclax-based (**G–L**) combinations with S63845 were obtained using the SynergyFinder+ R package. Plots were generated using data from 2D cell culture experiments in LNCaP95 (**A**, **D**, **G**, and **J**), VCaP-CR (**B**, **E**, **H**, and **K**), and 22Rv1 (**C**, **F**, **I**, and **L**). Data provided represent mean ± SD from three individual experiments.

We used the SynergyFinder+ R package (18) to calculate and visualize the Bliss synergy for the combination of Bcl-2 family–targeting inhibitors with S63845 utilizing a customized 6 × 8 matrix layout. The combination of A-1331852 and S63845 yielded the highest synergy scores of 24.59, 27.37, and 29.59 across LNCaP95, VCaP-CR, and 22Rv1 cell lines, respectively (Fig. 3A–F). The combination of navitoclax and S63845 followed with synergy scores of 17.63, 15.19, and 27.24 for the same three cell lines, respectively (Fig. 3G–L). Finally, venetoclax and S63845 had the lowest synergy scores of 12.96, 4.47, and 12.98 for LNCaP95, VCaP-CR, and 22Rv1 cell lines, respectively (Supplementary Fig. S3D–S3I). Overall, the combination of S63845 and A-1331852 was superior to S63845 paired with navitoclax or venetoclax.

### Co-inhibition of Mcl-1 and Bcl-xL induces apoptotic activity through PARP and caspase 3/7 cleavage

Next, we examined the effect of combined inhibition of Bcl-xL and Mcl-1 on several proteins of interest through Western blotting analysis. We treated our cell lines with single agents (at IC_50_) and combination doses highlighted by the Bliss synergy plots. Treatment with A-1331852 and S63845 led to modest changes in protein levels of full-length AR, AR-V7, and Bcl-xL in LNCaP95 and 22Rv1 cells (Supplementary Fig. S4). Notably, the combination of A-1331852 with S63845 led to increased levels of c-PARP at 24 hours after treatment, indicating the occurrence of apoptosis.

**Figure 4:**
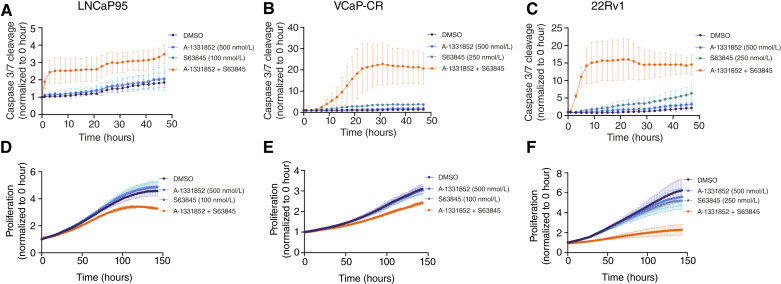
Live-cell imaging reveals dynamics of apoptosis and proliferation dynamics after dual treatment of A-1331852 and S63845. **A** to **C,** Count of cells with notable caspase 3/7 cleavage measured by a green fluorescent signal after dual treatment. **D** to **F,** Proliferation of cells after dual treatment as measured by the confluency of each well. All experiments contained three biological and three technical replicates. Data are plotted as mean ± SD of the means from each experiment.

We next investigated the dynamics of apoptosis induction for our combination treatment as well as how this affects proliferation of our cell lines up to 6 days after treatment. Notably, the co-treatment of A-1331852 with S63845 at our synergistic doses led to a rapid induction of apoptosis starting at 1 to 7 hours after treatment and lasting through 48 hours (Fig. 4A–C). Although apoptosis happened relatively soon after treatment, a difference in proliferation was not seen until 24 to 72 hours after treatment (Fig. 4D–F). Only our combination treatment led to differential levels of apoptosis and proliferation. Taken together, these results suggest that the synergy between A-1331852 and S63845 leads to a rapid induction of apoptosis and a lagged effect on proliferation in which there is no effect as single agents.

### Combination targeting of Bcl-xL and Mcl-1 reduces cell viability in 3D spheroids and organoid models

We next assessed the use of BH_3_ mimetics in CRPC spheroids which are more representative models of intercellular interactions, drug penetration, and physiologically relevant gradients (29). We compared dose responses in 2D and 3D to look for any major shifts in single-agent drug response between the two models. After 72 hours of spheroid formation, all BH_3_ mimetics displayed dose-dependent antiproliferative effects that were comparable with their activity ranges in 2D culture systems (Supplementary Fig. S5; Supplementary Table S2).

**Figure 5:**
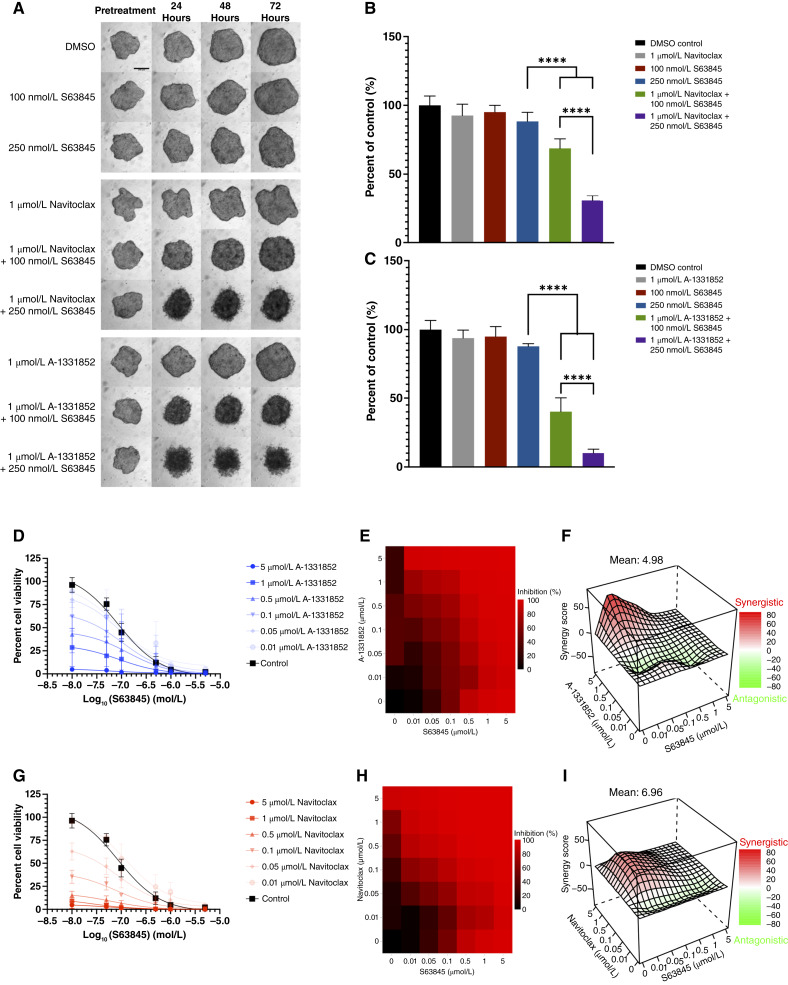
Dual targeting of Bcl-xL and Mcl-1 leads to synergistic decreases in cell viability in 3D LNCaP95 spheroids and the LuCaP 167CR organoid model. Dual treatment of navitoclax and A-1331852 with S63845 in 3D prostate cancer models. **A,** Representative images for each treatment condition before treatment and 24, 48, and 72 hours after treatment. **B** and **C,** Viability of spheroids measured by 3D CellTiter-Glo after 72 hours of treatment. Plots show mean ± SD. ****, *P* < 0.0001. LuCaP-167CR organoids were treated with the combination of A-1331852 and S63845 (**D–F**) or navitoclax and S63845 (**G–I**). Drug curves (**D** and **G**) show mean ± SD. Dose–response matrices (**E** and **H**) and Bliss synergy plots (**F** and **I**) were generated using the Synergyfinder+ R package to evaluate synergy. All spheroid experiments were completed in triplicate. Organoid experiments had six technical replicates with three biological replicates.

We then explored the co-targeting of Bcl-xL and Mcl-1 using CRPC spheroids. A dose of 1 μmol/L navitoclax/A-1331852, 100 nmol/L S63845, or 250 nmol/L S63845 led to minor reductions in cell viability with no visible effects on spheroid size and/or fraying (Fig. 5A–C; Supplementary Fig. S6). The combination of 1 μmol/L navitoclax or 1 μmol/L A-1331852 with 100 or 250 nmol/L S63845 led to significant decreases in cell viability across all three cell lines, with the exception of 1 μmol/L navitoclax and 100 nmol/L S63845 in VCaP-CR spheroids (Fig. 5A–C; Supplementary Fig. S6). Co-treatment with 1 μmol/L navitoclax/A-1331852 and 250 nmol/L S63845 led to visible spheroid fraying as early as 24 hours after treatment in LNCaP95 spheroids (Fig. 5A).

We then examined the effect of the same treatment combination (A-1331852 plus S63845) in the LuCaP 167CR prostate cancer organoid model. The parental LuCaP 167 model was derived from a liver metastasis of a patient resistant to docetaxel, carboplatin, and abiraterone (21, 22) with high AR-V7 expression and was primed for growth under castration-resistant settings. The model has PTEN loss, similar to LNCaP95 cells, as well as an Mcl-1 amplification (22). With increasing concentrations of each BH_3_ mimetic, either as a single agent or combination, a corresponding decrease in cell viability was observed (Fig. 5D–I).

The combinations of A-1331852 and navitoclax with S63845 resulted in a decrease in the IC_50_ of S63845 by up to 10-fold as the concentration of each BH_3_ mimetic increased (Fig. 5D–I; Supplementary Fig. S7). Synergy analysis performed using SynergyFinder+ showed a decreased synergy outcome relative to our 2D models (Fig. 5F and I) likely because of the increased potency of single-agent activity of the organoid model to S63845, A-1331852, and navitoclax. Both scores fell into the additive range; thus, we could not draw a conclusion on which combination was superior in our organoid model. The combination therapies effects on the more complex organoid models build upon our work in 3D spheroid and 2D models.

### XZ739, a Bcl-xL–targeting proteolysis-targeting chimera, synergizes with S63845, providing a potential route for avoiding toxicity

**Figure 6:**
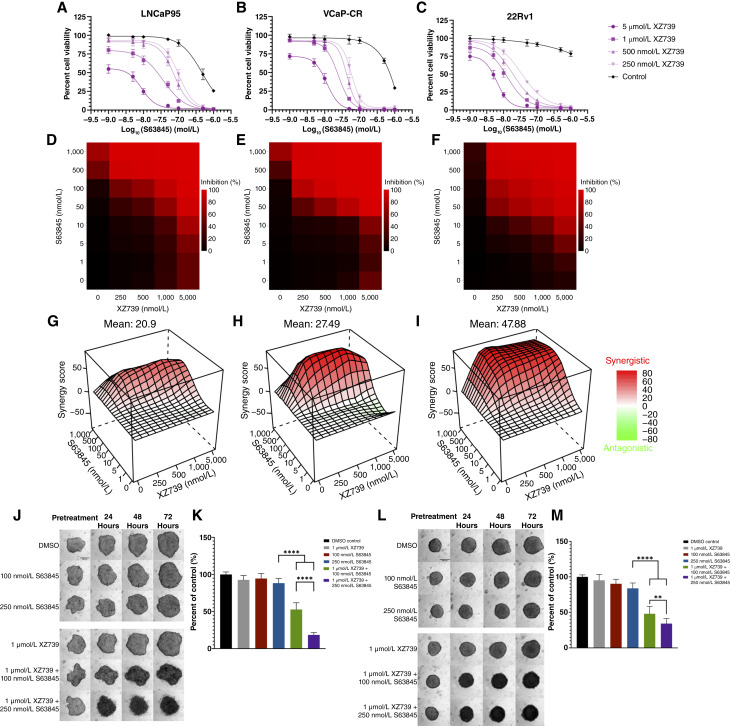
XZ739 in combination with S63845 has synergistic antitumor effects in CRPC cell lines. **A** to **I,** CellTiter-Glo viability data from 2D cultures of LNCaP95, VCaP-CR, and 22Rv1 cell lines. Cell viability data for the dual treatment (**A–C**) were generated by CellTiter-Glo. Dose–response matrices (**D–F**) and Bliss synergy plots (**G–I**) were created using the SynergyFinder+ R package. **J** to **M,** 3D spheroid model and cell viability of LNCaP95 (**J** and **K**) and 22Rv1 cells (**L** and **M**). Data are presented as mean ± SD. **, *P* < 0.01; ****, *P* < 0.0001. All experiments were completed in triplicate. Spheroid images are representative of three replicates.

Drugs targeting Bcl-xL have had limited clinical utility as monotherapy in solid tumors because of platelet toxicity as platelets require Bcl-xL to maintain viability (30). One approach to mitigate thrombocytopenia associated with Bcl-xL inhibition is through the use of Bcl-xL–targeted proteolysis targeting chimeras (PROTAC). XZ739, a Bcl-xL–targeting PROTAC, degrades Bcl-xL in a cereblon (CRBN)-dependent manner. Importantly, platelets express low levels of CRBN, so the use of a PROTAC may spare platelet toxicity (30). We next evaluated XZ739, a CRBN-dependent Bcl-xL degrader, that uses navitoclax as a “warhead” in combination with S63845. XZ739 was developed to overcome thrombocytopenia due to reduced CRBN E3 ligase expression in platelets (30, 31). Single-agent XZ739 treatment resulted in IC_50_ values greater than 10 μmol/L in our AR-V7–expressing prostate cancer lines (Supplementary Fig. S8). S63845’s potency increased when co-treated with XZ739, similar to the effect of co-treatment with navitoclax/A-1331852. Bliss synergy scores for XZ739 in combination with S63845 were 20.90, 27.49, and 47.88 for LNCaP95, VCaP-CR, and 22Rv1 cells, respectively (Fig. 6A–I). Similar to the co-treatment of A-1331852 or navitoclax with S63845, we observed synergistic activity between XZ739 and S63845. In addition, the treatment combination of XZ739 and S63845 led to a strong decrease in Bcl-xL levels. The co-treatment also led to a slight decrease in AR-V7 and Mcl-1 in 22Rv1 cells. Strikingly, co-treatment led to an increase in c-PARP levels in both cell lines (Supplementary Fig. S9). These results suggest that the Bcl-xL–degrading activity of XZ739 subsequently induces apoptosis.

Although single-agent XZ739 treatment had little effect on cell viability, co-treatment with XZ739 and S63845 led to synergistic decreases in cell viability (Fig. 6J–M) and visible spheroid border fraying as early as 24 hours after treatment (Fig. 6J–M). Taken together, these results suggest that XZ739 performs similarly to A-1331852 and navitoclax when paired with S63845.

## Discussion

Utilizing a high-throughput combination drug screen, we identified BH_3_ mimetics as potent and synergistic inhibitors of CRPC cell viability. Targeting the intrinsic apoptosis pathway with BH_3_ mimetics represents an attractive therapeutic strategy for patients with advanced prostate cancer (32). The current study aimed to validate and optimize a combination approach for patients with mCRPC who have developed resistance to all standard care. Our findings demonstrate that Bcl-xL and Mcl-1 co-targeting is the most efficacious combination, followed by the co-targeting of Bcl-2 and Mcl-1. These findings are intriguing from a clinical perspective as patient samples from the SU2C mCRPC cohort revealed differential expression in Bcl-2 family genes. In these cancer tissues, the expression of Mcl-1 was the highest, followed by Bcl-xL and Bcl-2, further justifying the co-targeting of Bcl-xL/Mcl-1 rather than Bcl-2/Mcl-1 (27). Additionally, prior studies focusing on parental LNCaP cells have also indicated the role of Bcl-xL upregulation in castration resistance (33) and the ability to indirectly target Mcl-1 to sensitize cells to navitoclax but not venetoclax (14).

Notably, our cell lines span many common alterations found in mCRPC. We previously sequenced these cell lines to determine the mutational status in key pathways (34). All cell lines used express AR-V7, a key biomarker indicating resistance to second-generation AR inhibitors in patients with mCRPC (2, 35, 36). LNCaP95 cells are PTEN deficient. 22Rv1 cells express other AR splice variants, as well as a heterozygous missense mutation in TP53 (Q331R; ref. 37). Lastly, VCaP-CR cells exhibit the TMPRSS–ERG fusion which is found in over half of patients with prostate cancer (38), as well as AR-FL amplification through AR copy-number gain (39, 40). Importantly, targeted Bcl-xL/Mcl-1 inhibition is efficacious across all models, even given their genomic differences.

We also investigated the efficacy of these combination treatments in prostate cancer organoids, which have heterogeneity more representative of patient tumors. Here, we used the LuCaP 167CR adenocarcinoma model. This model was derived from the LuCaP 167 model which is similar to the LNCaP95 cell line in that it has loss of PTEN and AR-V7 expression (41). Previous studies have shown upregulation of AR-V7 following castration in the LuCaP 167 model (41, 42). The LuCaP 167CR organoid model had enhanced sensitivity to S63845 as a single agent. This could be due to Mcl-1 copy-number gain observed in the parental LuCaP 167 model (22). We also demonstrated the sensitivity of organoid cultures to the combination treatments of navitoclax and S63845, as well as A-1331852 and S63845. These findings further support future studies of these combinations and emphasize the value of utilizing organoid cultures in validating drug screens.

Co-dependency on Mcl-1 and Bcl-xL for cell survival is a trend observed in several solid tumors in contrast to hematologic malignancies that are more dependent on Bcl-2 (43). Prior studies in cervical cancer, melanoma, medulloblastoma, head and neck squamous cell carcinoma, and pancreatic cancer have evaluated A-1331852, navitoclax, and/or venetoclax in combination with S63845, showing similar improvements in efficacy when co-targeting Bcl-xL and Mcl-1 over Bcl-2 and Mcl-1 (11–13, 44, 45). In medulloblastoma, WEHI-539 (Bcl-xL inhibitor) combined with S63845 was superior to venetoclax and S63845 in synergistically reducing cell viability (13). Although it has been suggested that differential binding affinities of the pro-apoptotic proteins, such as BAK (binds Bcl-xL, Bcl-2, and Mcl-1) and BAX (only binds Bcl-xL and Mcl-1), could explain differences in activity between the drug combinations, knockout of both BAX and BAK in melanoma had a similar influence on all BH_3_-mimetic combinations (12). This recent study found efficacy in targeting Bcl-xL, Bcl-2, and Mcl-1 across their models, whereas we found dual targeting of Bcl-xL and Mcl-1 (through dual treatment with A-1331852/S63845) to be the most efficacious.

Although BH_3_-mimetic drug combinations are highly efficacious, toxicity remains a critical concern when considering clinical development. Navitoclax has seen limited progress in the clinic because of severe dose-limiting thrombocytopenia (46, 47). A recent phase I/II clinical trial in solid tumors that utilized a 7-day lead in the dosing schedule of navitoclax (at a smaller dose) did not observe this toxicity (48). Mcl-1 inhibition has been associated with cardiotoxicity and adverse effects on both hematopoietic and lymphoid cells (49–51). Thus far, two studies have completed animal studies using the combination of A-1331852 and S63845. One study found acute liver toxicity in lung squamous cell carcinoma–xenografted NSG mice (52), whereas the other study used melanoma-xenografted nude mice treated at lower doses, avoiding this toxicity while demonstrating efficacy (53).

The development of combined and direct Bcl-xL and Mcl-1 inhibition proves to be complex because of toxicity seen in both the preclinical and clinical settings. Besides exploring lower doses of BH_3_ mimetics, we explored the use of a PROTAC Bcl-xL–specific degrader, XZ739, which binds to CRBN, a protein lowly expressed in platelets, and mediates the targeted degradation of Bcl-xL. XZ739 is 20-fold more potent than navitoclax, and it has less platelet toxicity (30). We found a similar potency of XZ739 to A-1331852 and navitoclax when combined with S63845 in our 2D models and our 3D tumor spheroids. Studies are ongoing to further evaluate XZ739 and other novel Bcl-xL PROTACs in combination with S63845 for use in *in vivo* prostate cancer models. The use of a CDK9 or mTORC1/2 inhibitor can indirectly inhibit Mcl-1 levels while potentially preventing cardiotoxicity. In acute myeloid leukemia cell lines, venetoclax combined with alvocidib (CDK9 inhibitor) reduced cell viability while reducing Mcl-1 levels (54). The combination of DT2216, a Bcl-xL–targeting PROTAC, and ADZ8055 (mTORC1/2 inhibitor) synergistically killed small cell lung cancer cells but not normal cells in mice (55). The use of PROTACs and indirect inhibitors of Bcl-2 family proteins may provide fewer off-target effects, leading to a more practical clinical combination.

This study is limited by the use of only AR-V7–expressing models although a recent study has shown synergy between navitoclax/S63845 dual treatment in a wide array of prostate cancer cell lines, including several that do not express AR-V7 (56). More mechanistic studies need to be completed to further our understanding of this combination. Future *in vivo* testing needs to be done on this combination to evaluate safety. Specifically, this should be done using alternative methods to direct co-targeting (e.g., indirect inhibition, PROTACs, or Mcl-1/Bcl-xL payload-based antibody–drug conjugates, etc.) to minimize potential toxicity. Co-targeting Bcl-xL and Mcl-1 shows synergistic activity in AR-V7–expressing CRPC models. Further research is needed to aid the continued development of BH_3_-mimetic drug combinations for the treatment of patients with mCRPC.

## Supplementary Material

Figure S1 Figure S1 shows the protein levels of AR, AR-V7, Bcl-xL, Mcl-1, and Bcl-2 across several prostate cancer cell lines.

Figure S2 Figure S2 shows the single agent activity of BH3 mimetics across LNCaP95, VCaP-CR, and 22Rv1 cells.

Figure S3 Figure S3 shows the effects of co-treatment of venetoclax with S63845 across 2D culture.

Figure S4 Figure S4 shows protein level changes after 24 hours of treatment with DMSO, A-1331852, S63845, or the combination of A-1331852 and S63845.

Figure S5 Figure S5 shows that A-1331852, Navitoclax, and S63845 have similar potency across 2D culture and 3D spheroids.

Figure S6 Figure S6 shows the effect of treamtent of 3D spheroids with BH3 mimetics as single agents or in combination.

Figure S7 Figure S7 depicts morphological changes of the LuCaP-167CR organoids after treatment with BH3 mimetics as single agents or in combination.

Figure S8 Figure S8 depicts the single agent activity of XZ739 across 2D culture.

Figure S9 Figure S9 depicts protein level changes after 24 hours of treatment with XZ739 alone or in combination with S63845.

Table S1 Table S1 depicts the IC50 values of BH3 mimetics as single agents in 2D models.

Table S2 Table S2 compares the IC50 values of BH3 mimetics as single agents across 2D culture and 3D spheroids.
