# Supplementary figures and images for: Direct Co-Targeting of Bcl-xL and Mcl-1 Exhibits Synergistic Effects in AR-V7–Expressing CRPC Models
Source: Cancer Res Commun. 2025 Aug 21;5(8):1396–408. doi: 10.1158/2767-9764.CRC-25-0096 (PMC12368576; doi:10.1158/2767-9764.CRC-25-0096)

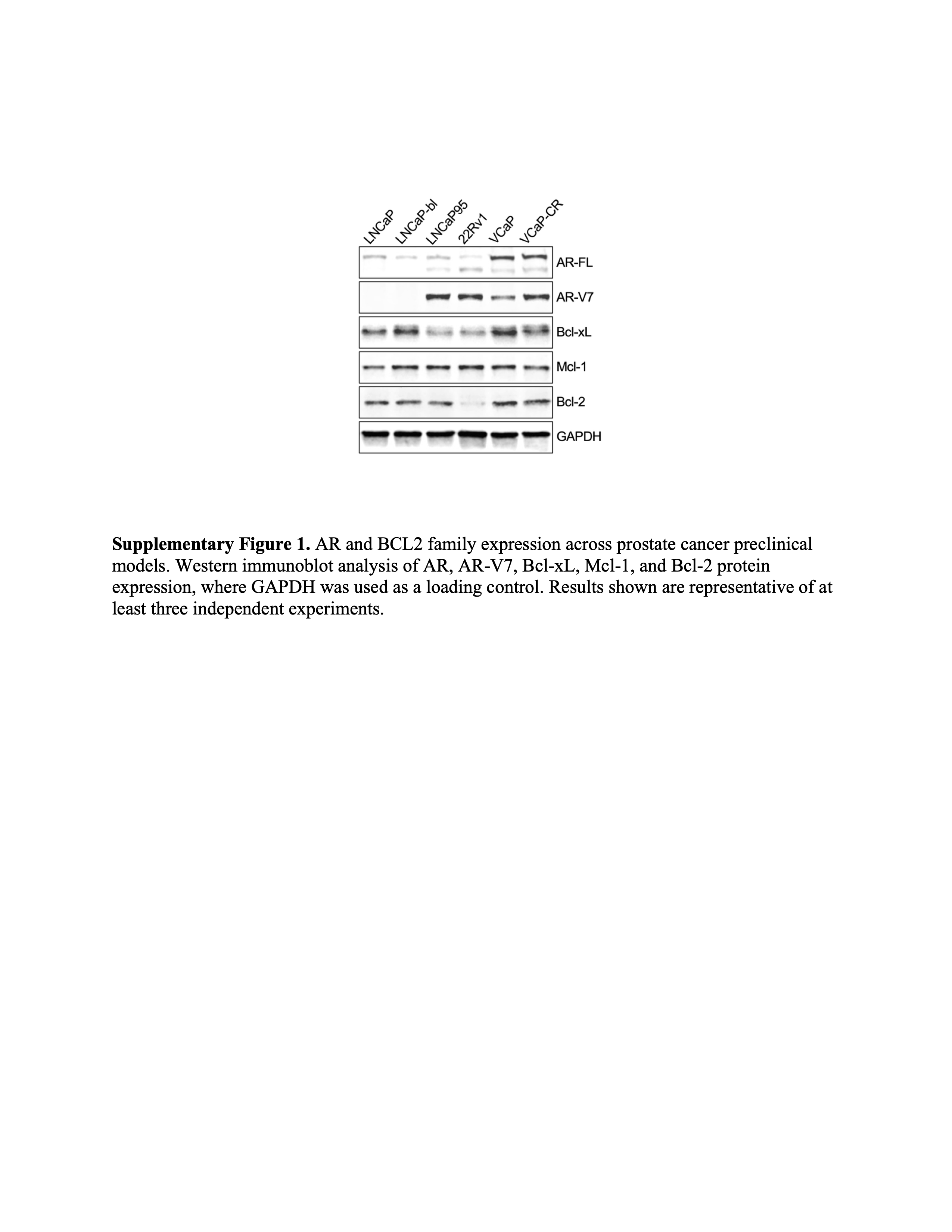

Supplement: Figure S1 — shows the protein levels of AR, AR-V7, Bcl-xL, Mcl-1, and Bcl-2 across several prostate cancer cell lines. [file crc-25-0096_figure_s1_suppsf1.png]

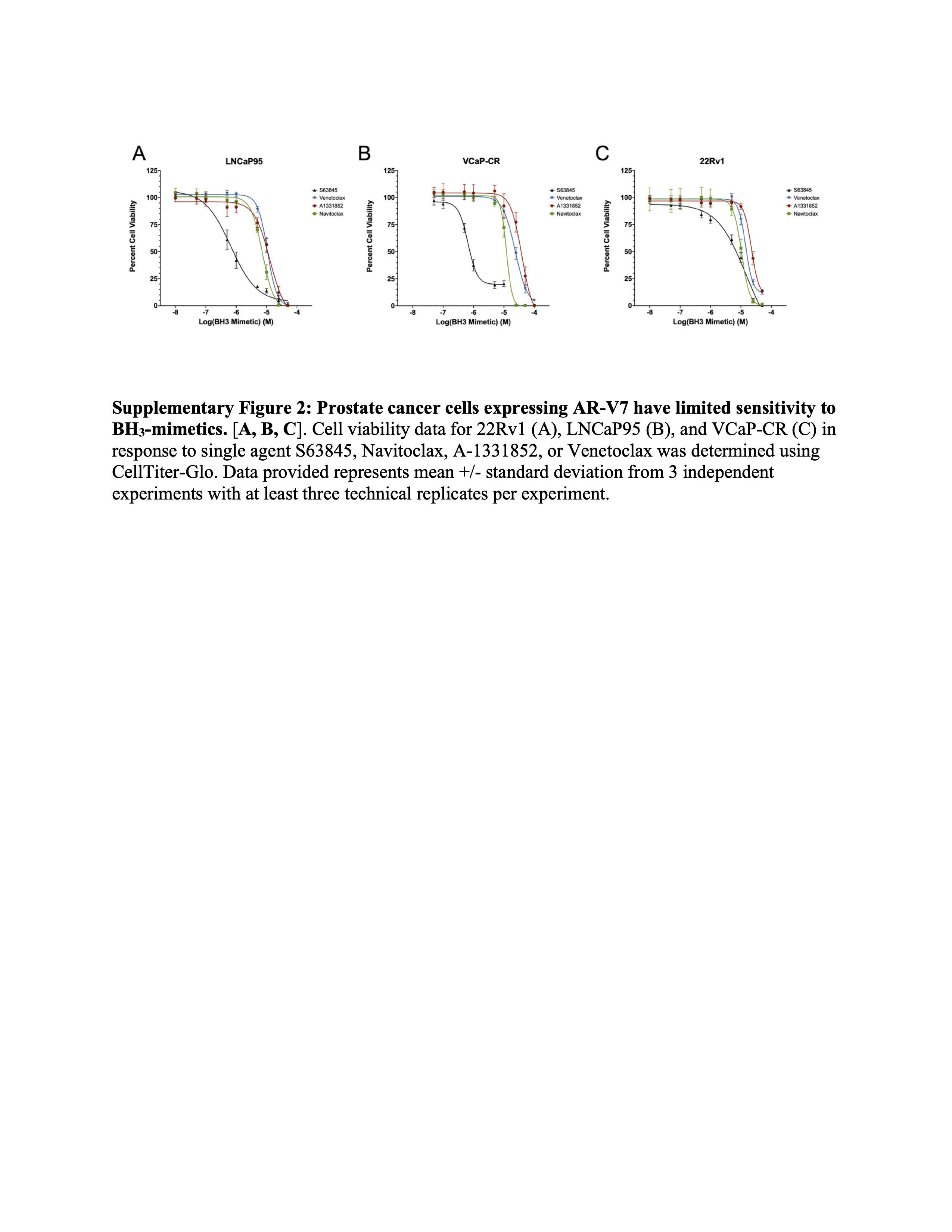

Supplement: Figure S2 — shows the single agent activity of BH3 mimetics across LNCaP95, VCaP-CR, and 22Rv1 cells. [file crc-25-0096_figure_s2_suppsf2.png]

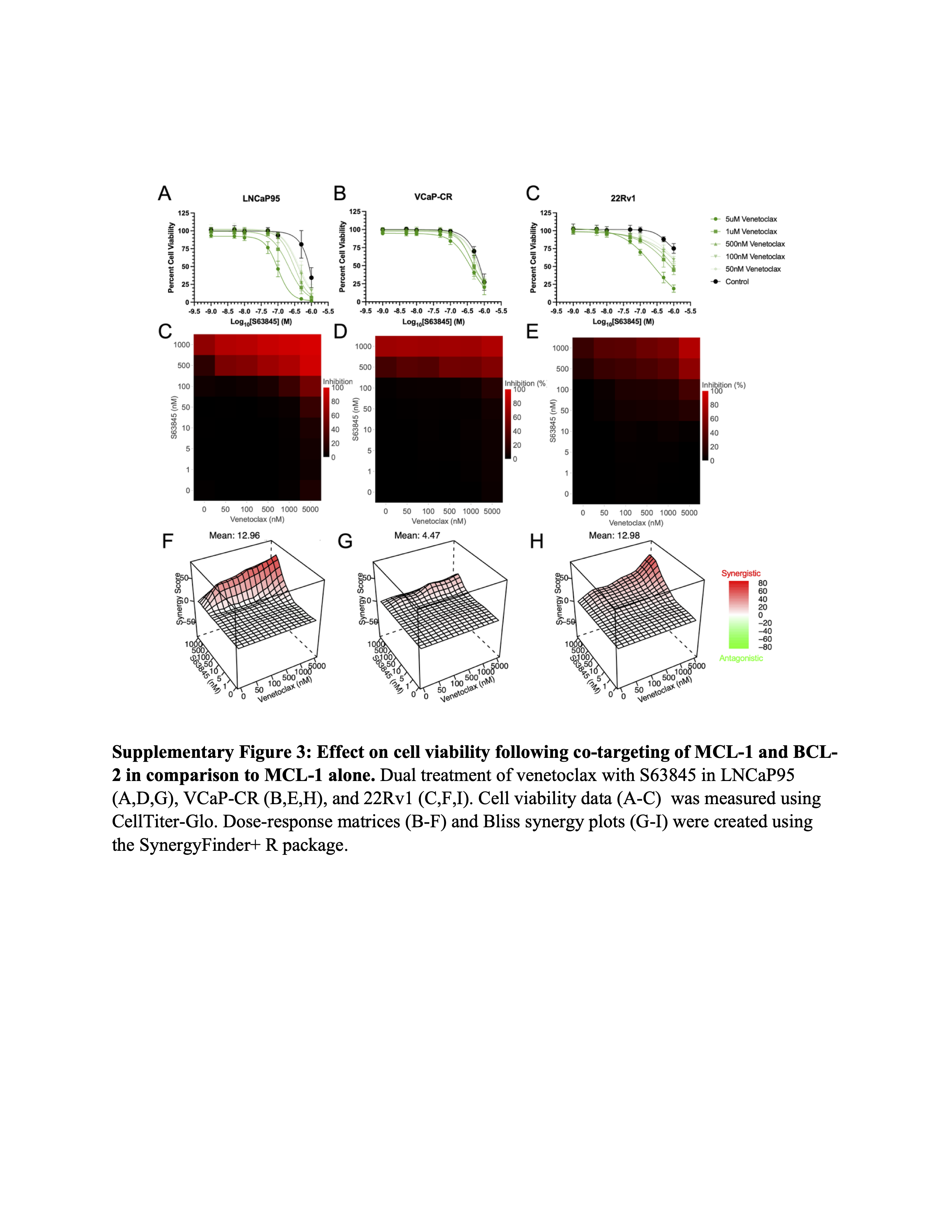

Supplement: Figure S3 — shows the effects of co-treatment of venetoclax with S63845 across 2D culture. [file crc-25-0096_figure_s3_suppsf3.png]

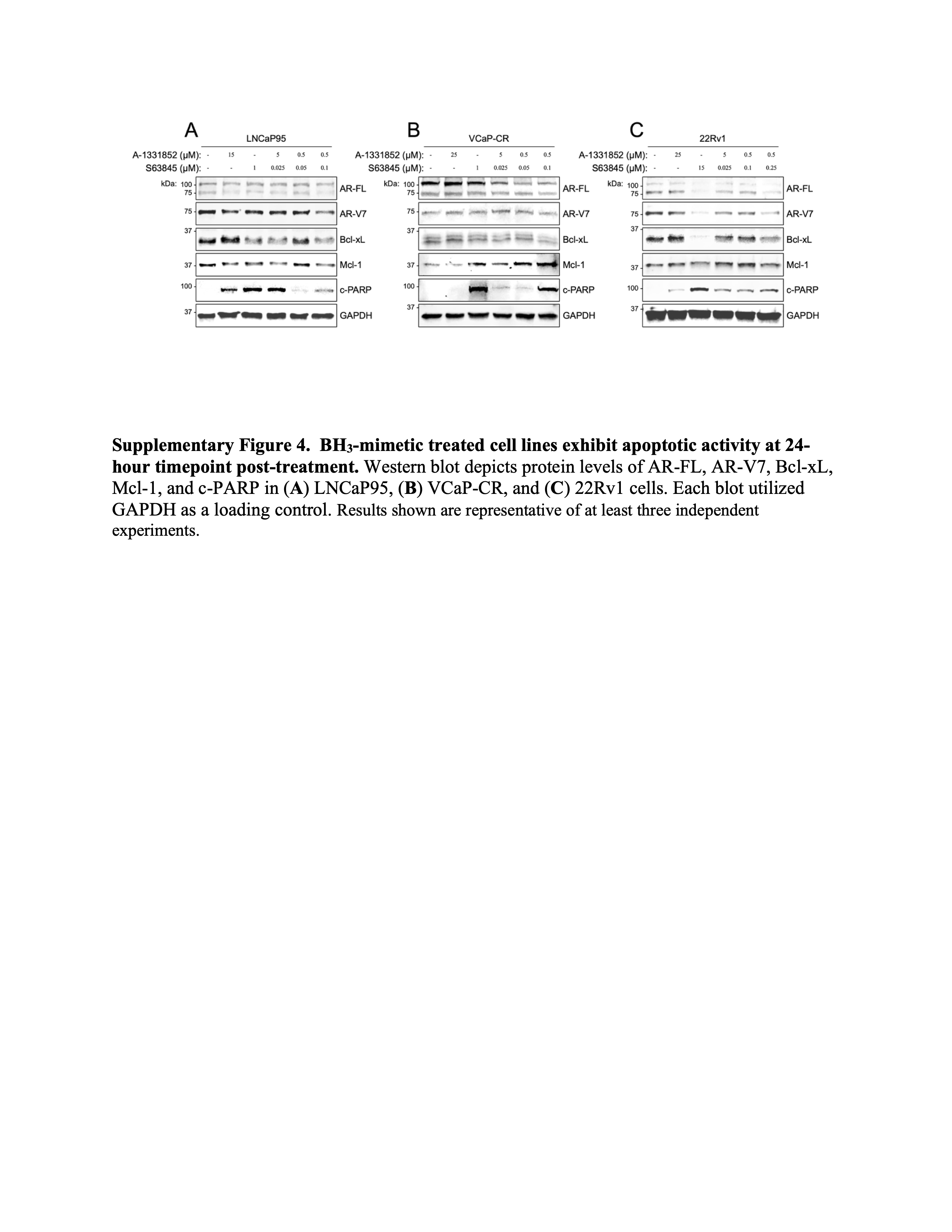

Supplement: Figure S4 — shows protein level changes after 24 hours of treatment with DMSO, A-1331852, S63845, or the combination of A-1331852 and S63845. [file crc-25-0096_figure_s4_suppsf4.png]

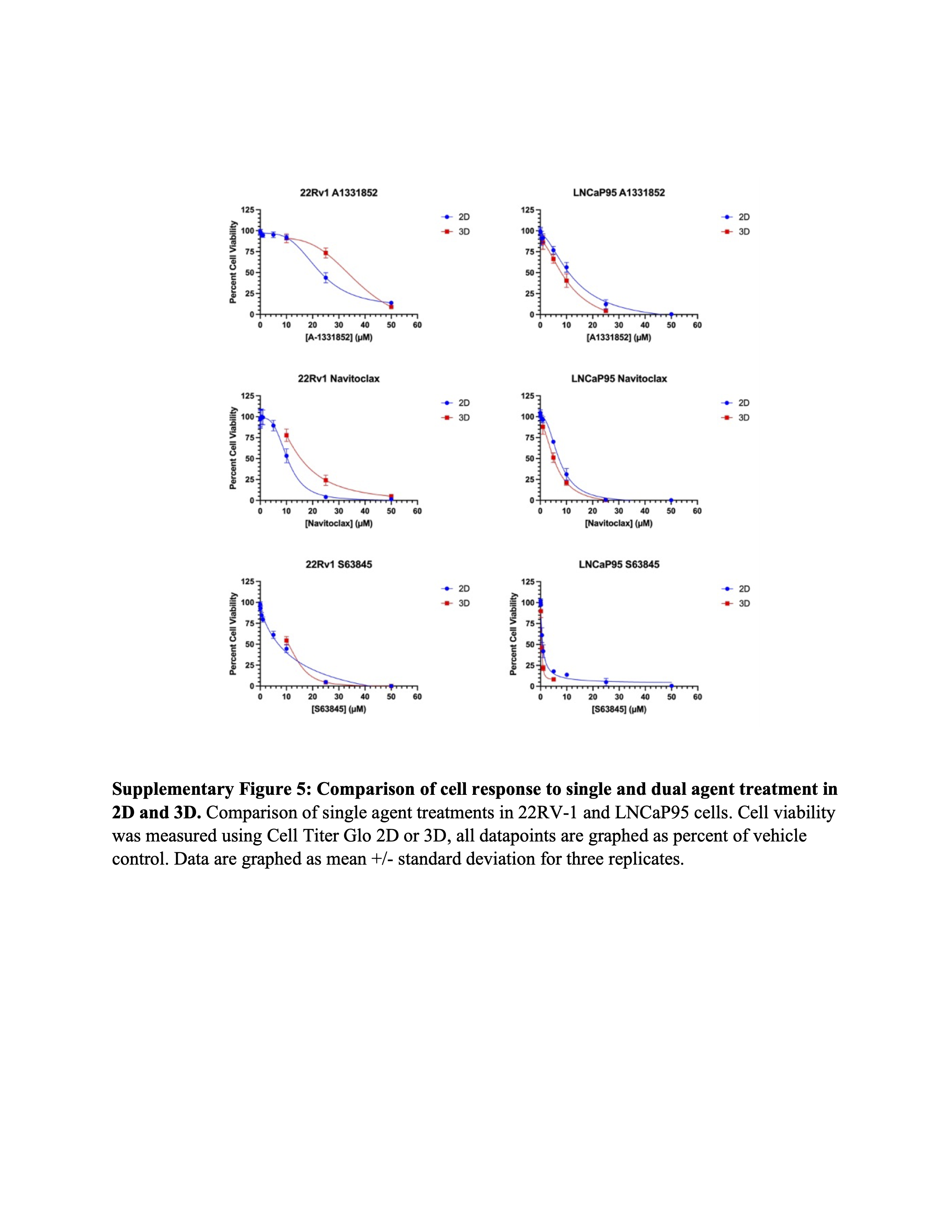

Supplement: Figure S5 — shows that A-1331852, Navitoclax, and S63845 have similar potency across 2D culture and 3D spheroids. [file crc-25-0096_figure_s5_suppsf5.png]

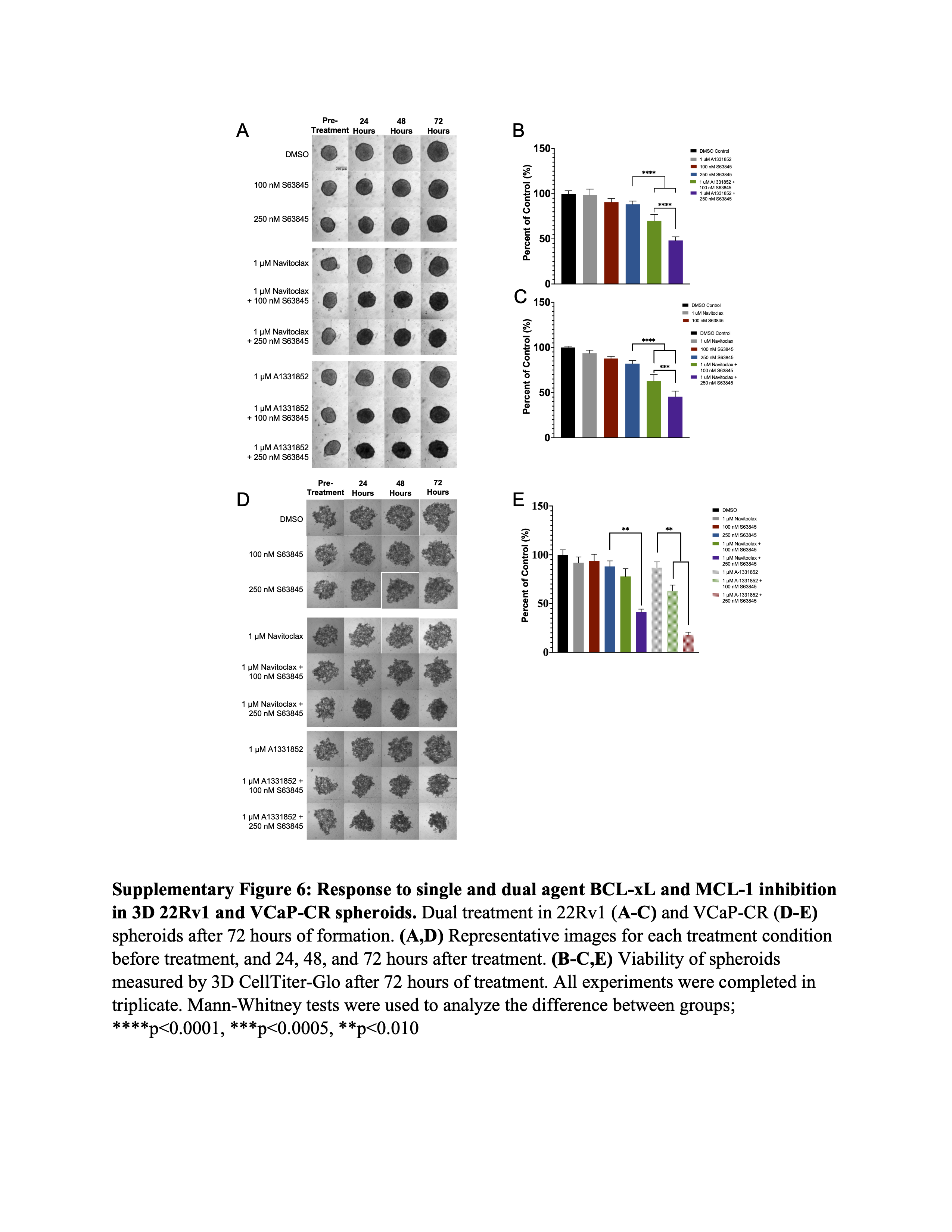

Supplement: Figure S6 — shows the effect of treamtent of 3D spheroids with BH3 mimetics as single agents or in combination. [file crc-25-0096_figure_s6_suppsf6.png]

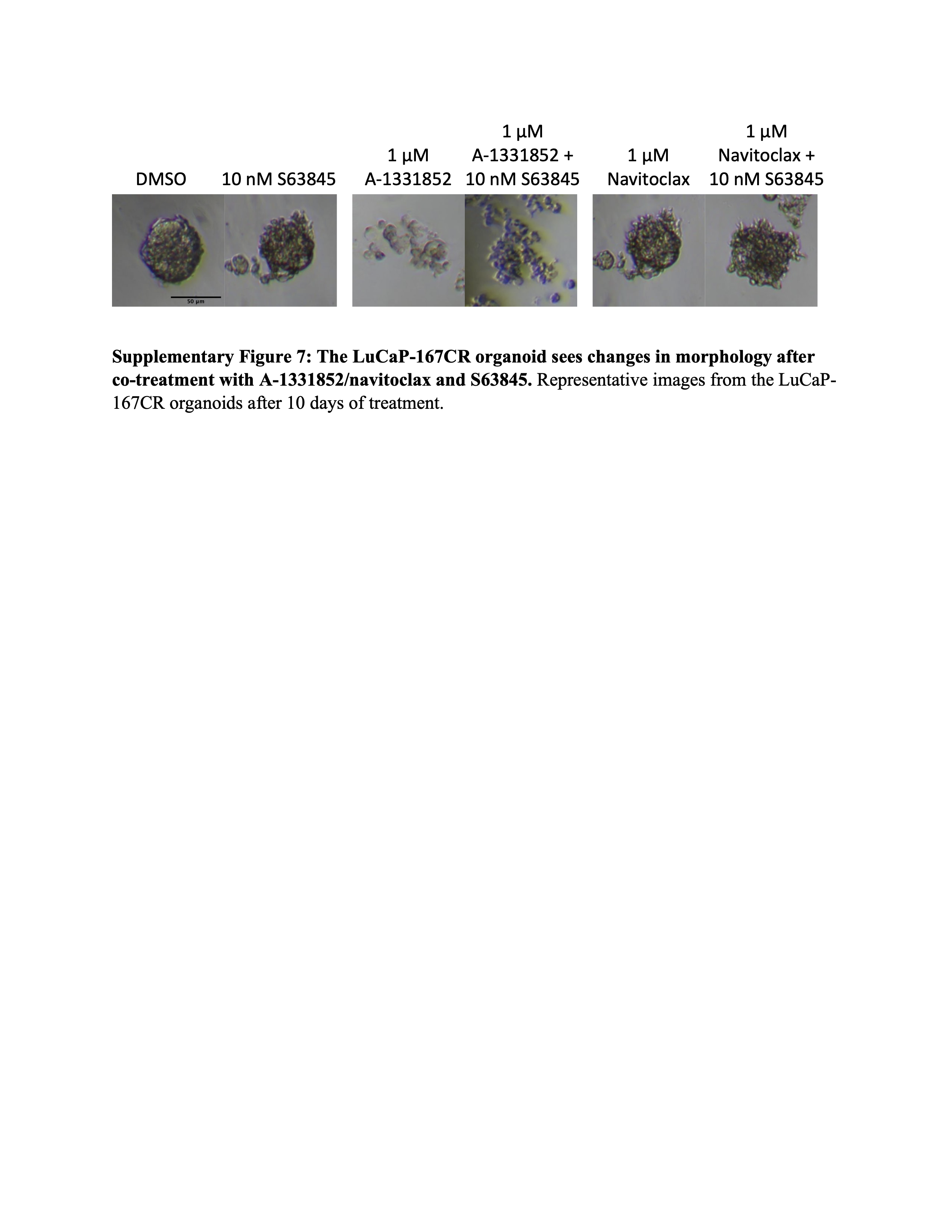

Supplement: Figure S7 — depicts morphological changes of the LuCaP-167CR organoids after treatment with BH3 mimetics as single agents or in combination. [file crc-25-0096_figure_s7_suppsf7.png]

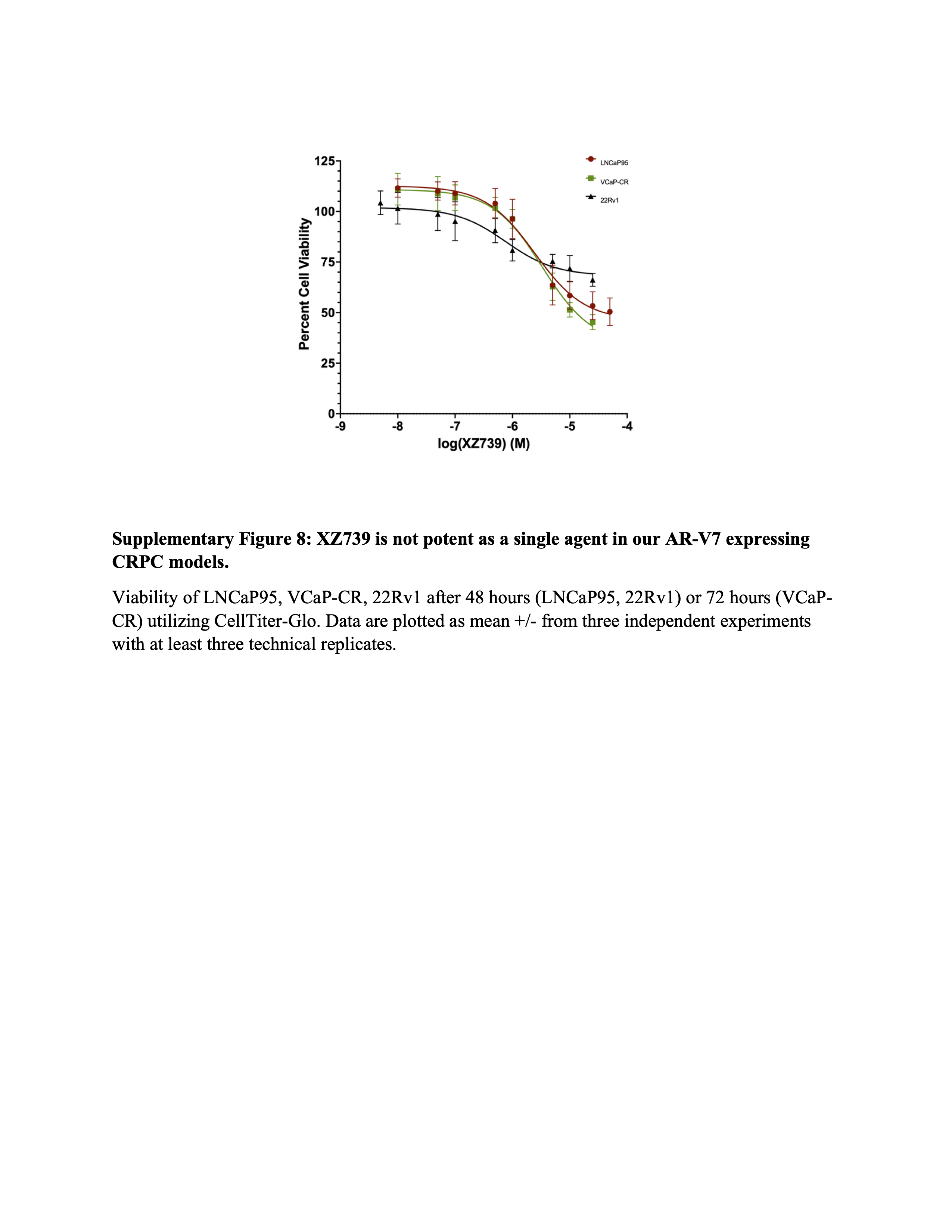

Supplement: Figure S8 — depicts the single agent activity of XZ739 across 2D culture. [file crc-25-0096_figure_s8_suppsf8.png]

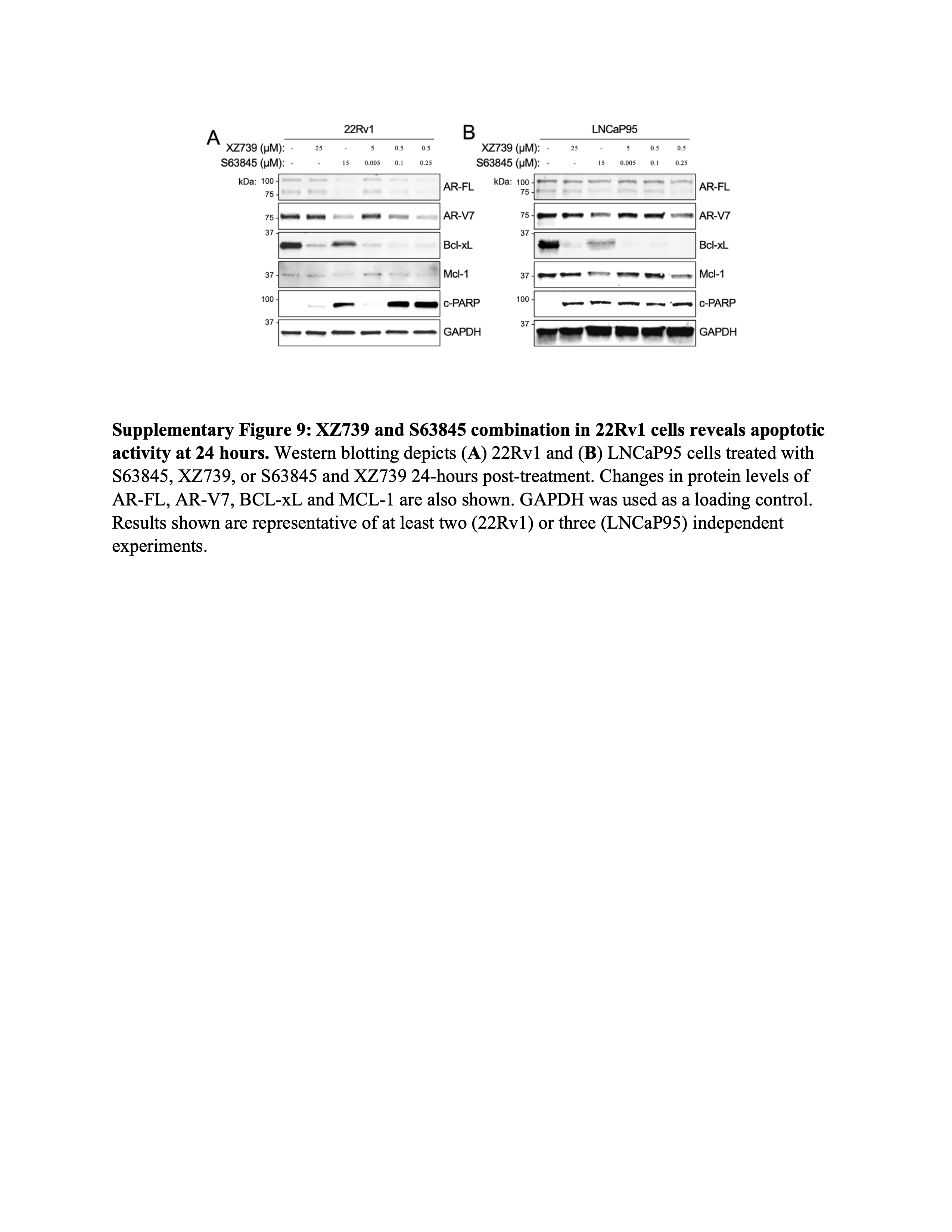

Supplement: Figure S9 — depicts protein level changes after 24 hours of treatment with XZ739 alone or in combination with S63845. [file crc-25-0096_figure_s9_suppsf9.png]
