# Supplementary material for: Direct Co-Targeting of Bcl-xL and Mcl-1 Exhibits Synergistic Effects in AR-V7–Expressing CRPC Models
Source: Cancer Res Commun. 2025 Aug 21;5(8):1396–408. doi: 10.1158/2767-9764.CRC-25-0096 (PMC12368576; doi:10.1158/2767-9764.CRC-25-0096)
Supplement: Table S1 — depicts the IC50 values of BH3 mimetics as single agents in 2D models. [file crc-25-0096_table_s1_suppst1.pdf]

Drug (Target)	LNCaP95 IC50 (µM)	VCaP-CR IC50 (µM)	22Rv1 IC50 (µM)
A-1331852 (Bcl-xL)	12.77	38.75	22.19
Navitoclax (Bcl-xL/2)	7.25	12.10	10.46
Venetoclax (Bcl-2)	10.60	23.62	14.29
S63845 (Mcl-1)	0.67	0.66	14.79

Supplementary Table 1: IC50 values of single agent BH3-mimetics *in vitro*. IC50 was generated from Graphpad Prism version by the log(inhibitor) vs. response – variable slope (four parameters) equation. Data is from three technical and three biological replicates.
