# Supplementary material for: Direct Co-Targeting of Bcl-xL and Mcl-1 Exhibits Synergistic Effects in AR-V7–Expressing CRPC Models
Source: Cancer Res Commun. 2025 Aug 21;5(8):1396–408. doi: 10.1158/2767-9764.CRC-25-0096 (PMC12368576; doi:10.1158/2767-9764.CRC-25-0096)
Supplement: Table S2 — compares the IC50 values of BH3 mimetics as single agents across 2D culture and 3D spheroids. [file crc-25-0096_table_s2_suppst2.pdf]

Drug (Target)	LNCaP95 IC50 2D (µM)	LNCaP95 IC50 3D (µM)	22Rv1 IC50 2D (µM)	22Rv1 IC50 3D (µM)
A-1331852 (Bcl-xL)	12.77	11.36	22.19	37.7
Navitoclax (Bcl-xL/2)	7.25	6.04	10.46	12.9
S63845 (Mcl-1)	0.67	0.45	14.79	13.25

Supplementary Table 2: IC50 values of BH3-mimetics in 2D and 3D models. IC50 was generated from GraphPad Prism by the [inhibitor] vs. response – variable slope (four parameters) equation. Data is from three technical and three biological replicates.
